# A Thermosiphon Photobioreactor for Photofermentative Hydrogen Production by *Rhodopseudomonas palustris*

**DOI:** 10.3390/bioengineering9080344

**Published:** 2022-07-27

**Authors:** Catharine Elizabeth Bosman, Robert William McClelland Pott, Steven Martin Bradshaw

**Affiliations:** Department of Process Engineering, Stellenbosch University, Banghoek Road, Stellenbosch 7600, South Africa; 18218962@sun.ac.za (C.E.B.); smb@sun.ac.za (S.M.B.)

**Keywords:** biohydrogen, thermosiphon photobioreactor, *Rhodopseudomonas palustris*, photofermentation

## Abstract

A thermosiphon photobioreactor (TPBR) can potentially be used for biohydrogen production, circumventing the requirement for external mixing energy inputs. In this study, a TPBR is evaluated for photofermentative hydrogen production by *Rhodopseudomonas palustris* (*R. palustris*). Experiments were conducted in a TPBR, and response surface methodology (RSM), varying biomass concentration, and light intensity and temperature were employed to determine the operating conditions for the enhancement of both hydrogen production as well as biomass suspension. Biomass concentration was found to have had the most pronounced effect on both hydrogen production as well as biomass suspension. RSM models predicted maximum specific hydrogen production rates of 0.17 mol m^−3^h^−1^ and 0.21 mmol g_CDW_^−1^h^−1^ at *R. palustris* concentrations of 1.21 and 0.4 g L^−1^, respectively. The experimentally measured hydrogen yield was in the range of 45 to 77% (±3.8%), and the glycerol consumption was 8 to 19% (±0.48). At a biomass concentration of 0.40 g L^−1^, the highest percentage of biomass (72.3%), was predicted to remain in suspension in the TPBR. Collectively, the proposed novel photobioreactor was shown to produce hydrogen as well as passively circulate biomass.

## 1. Introduction

Hydrogen has gained increasing interest as a potential energy carrier [1]. Moving in the direction of sustainability, some biological processes currently being investigated for hydrogen production include microbial photofermentation and dark fermentation [1], which employs suitable microorganisms to convert renewable substrates, such as waste streams, to biohydrogen, in a clean, non-polluting manner. This offers a promising circular economics approach for sustainable hydrogen production, as well as responsible waste management [2]. Another biological process also currently being investigated is bio-photolysis using microalgae (cyanobacteria and green/blue-green algae)—a process in which the microorganisms are used to photosynthetically split water molecules into hydrogen and oxygen [3].

Dark fermentation has been widely investigated and proven to be the most suitable method for sustainable biohydrogen production [3,4]. Dark fermentation is the process in which suitable microorganisms are used to generate hydrogen gas from suitable carbon substrates under anaerobic conditions and in the absence of a light source [5]. This method of biohydrogen production has several advantages over other methods—no need for light energy, with the productivity not being limited by the presence of oxygen, and the system being carbon neutral as well as the possibility of using carbon-rich waste streams as a substrate [5]. Nonetheless, this method also has some disadvantages—a low conversion efficiency of carbon to hydrogen, as well as the production of volatile fatty acids as by-products [5]. Conversely, photofermentation is known for its high substrate-to-hydrogen conversion efficiencies, but slightly lower production rates [5,6]. The main differences between dark and photofermentation are the presence of light energy in photofermentation, and dark fermentation being facilitated by the hydrogenase enzyme, while photofermentation is primarily facilitated by the nitrogenase enzyme. Purple non-sulfur bacteria have been identified as an attractive prospect for photofermentative hydrogen production, due to their high substrate-to-hydrogen conversion efficiency [7,8] and their potential for the bioremediation of waste streams [9]. The species *R. palustris* shows great promise for photofermentative hydrogen production, due to its acclimation ability to light intensity [10,11] and temperature [12]. Hydrogen production by *R. palustris* is principally facilitated by the photoheterotrophic metabolic route, meaning metabolism in the presence of light, a suitable carbon substrate, and under an anaerobic atmosphere. However, strain-dependent *R. palustris* has been shown to produce hydrogen in the temperature range of 30 to 42 °C, with 42 °C also being its physiological upper limit, beyond which the bacterial cells start to die [12]. *R. palustris* is not associated with photoinhibition, due to it not being an oxygen-evolving microorganism, and also due to its ability to dissipate excess energy from high light intensities as heat through the use of carotenoids [13]; however, productivity has been shown to decrease beyond light intensities of approximately 500–600 W m^−2^ [6,14]. Under the right conditions, *R. palustris* cells have been shown to achieve hydrogen production rates of up to 1.96 mol m^−3^h^−1^ [15]. Under outdoor conditions, *R. palustris* has been shown to achieve hydrogen production rates of up to 1.2 mol m^−3^h^−1^ [16], while other purple non-sulfur bacteria have shown similar rates (*Rhodobacteror capsulatus*, 0.31–1.3 mol m^−3^h^−1^ [17,18,19,20,21,22,23,24]; *Rhodobacter spaeroides*, 0.45 mol m^−3^h^−1^ [25,26]; *Rhodopseudomonas spaeroides*, up to 4.5 mol m^−3^h^−1^ [27]).

At present, the energy requirements for biohydrogen production are still predominantly greater than the energy output of the hydrogen product [16,28]. A recent techno-economic analysis on photofermentative hydrogen production from sugar beet molasses reported hydrogen costs of around 33 EUR/kg—substantially more than the cost-level price of fossil fuel hydrogen (<2.7 EUR/kg in Germany and the United States), water electrolysis hydrogen using renewable energy (<3.2 EUR/kg) [28], as well as some of the green hydrogen retail prices currently being reported by ongoing projects around the world (around 2.8 to 5 EUR/kg) [29]. To circumvent these high operating costs, three main strategies are currently being implemented: the use of natural means of mixing [30,31], solar radiation [16,32], and the use of industrial waste streams as a carbon substrate, concurrently treating these waste streams [7,9,33,34]—with numerous studies being conducted on the latter two strategies, while natural mixing in photobioreactors (PBRs) has received comparatively little attention. Continuous mixing is an important factor in photobioreactor design. It not only allows bacterial cells to be exposed to the light, eliminating dark zones, but it also allows enhanced contact with nutrients in the reactor medium. The combination of enhanced light exposure and mass transfer increases hydrogen productivity—the ultimate aim of photofermentative photobioreactors.

Crude glycerol, a by-product of the biodiesel industry, has gained increasing attention as a substrate in biological waste to value-added product processes—some of these processes include biogas production by anaerobic digestion [35,36,37], lipid production [38], and photofermentative hydrogen production [7], with Pott et al. [7] reporting a conversion efficiency (crude glycerol to hydrogen gas) that is close to 90% of the theoretical maximum when using *R. palustris*. The combination of industrial waste streams as a carbon substrate and a cost-efficient PBR would not only decrease the cost of photofermentative hydrogen production [34], but also aid in moving towards sustainable hydrogen production and green energy.

One PBR implementing natural mixing is the thermosiphon photobioreactor (TPBR) [39]. Such a TPBR would ideally require no mixing energy input, as it utilizes a temperature-induced density difference through heating (illumination) on one side and cooling on the opposite side, to drive circulation around the reactor [30]. The constant temperature differential causes quiescent conduction, and under conditions allowing for a sufficiently large temperature gradient, buoyancy-driven convection [40]. The heated low-density fluid rises to the top of the TPBR while simultaneously being displaced by the descending cooled high-density fluid [40,41]. Ultimately, this process results in the natural circulation of biomass, circumventing the requirement for external mixing and its concomitant costs [39,41]. When operating under natural sunlight and implementing a cooling strategy not requiring energy input, e.g., cooling fins, such a TPBR will have the ability to operate without any external energy inputs.

Recently, a study on a prototype TPBR reported a promising ability for the circulation of biomass, where the TPBR (1 L working volume) was able to maintain up to 83% of active *R. palustris* (NCIMB 11774) cells (concentration of 0.5 g L^−1^) in free suspension over a period of 4 h [30]. Using computational fluid dynamics simulations, an estimated maximum fluid velocity of 0.069 m s^−1^ was reported [30] which falls well within the range of the literature-reported fluid velocities of more conventional PBRs such as the wholly (0.038 m s^−1^), half (0.11 m s^−1^), and alternately (0.36 m s^−1^) aerated airlift PBRs [42] and tubular PBRs (0.2 m s^−1^) [16]. However, to date, no research has been conducted on the effect of critical individual parameters and the interaction of parameters affecting the circulation of biomass in such a TPBR, and the TPBR is yet to be implemented for photofermentative hydrogen production. For passive circulation to occur in the TPBR, it is imperative to investigate parameters such as the light intensity provided to the heated side of the reactor, as well as the cooling on the opposite side, as these parameters drive circulation. Under artificial illumination (150 W halogen lamps), a temperature differential of approximately 5 °C between the riser and downcomer sections of the TPBR was reported when operating in a temperature range of approximately 39.6 to 44.7 °C [43]. To add to the complexity, these ranges for light intensity and temperature do not necessarily coincide with the physiological ranges within which *R. palustris* can optimally produce hydrogen. Consequently, for *R. palustris* to be able to produce hydrogen in a TPBR, insight is required into the effect of these conditions.

The overall research hypothesis of this study is that a novel thermosiphon photobioreactor using passive circulation, can be implemented for the application of photofermentative hydrogen production by *R. palustris* under suitable operating conditions. Response surface methodology is applied to systematically determine the effect of some of the most critical factors, such as light intensity, temperature, and biomass concentration, affecting photofermentative hydrogen production as well as biomass circulation in a thermosiphon photobioreactor. In addition, suggestions are also made on operating conditions to enhance hydrogen production as well as biomass suspension in a TPBR, adding to the understanding and development of more cost-effective photobioreactors for sustainable hydrogen production.

## 2. Materials and Methods

### 2.1. Bacterial Strain and Culturing

The purple non-sulfur bacterium *Rhodopseudomonas palustris* strain NCIMB 11774 was used for this study. *R. palustris* cells were precultured in a fast-growing Van Niels medium (ATCC^®^ medium 112) containing (per liter): 1 g K_2_HPO_4_, 0.5 g MgSO_4_, 10 g yeast extract and filled up with deionised water [12]. The medium was autoclaved at 121 °C for 20 min, after which 10 mL of 4 M glycerol, autoclaved separately, was aseptically added to the 1 L medium [12]. Bacterial cells were resuspended in the medium and grown anaerobically in 500 mL Schott bottles, under an argon atmosphere. The culturing temperature was maintained at 35 °C (±0.2 °C) using a water bath, and illumination was provided by 100 W incandescent light bulbs (Eurolux ©, Milnerton, South Africa). Light intensity was calibrated at approximately 200 W m^−2^ (±20 W m^−2^) in the spectral range of 500 to 1100 nm. The cultures were allowed to grow for five days up until the mid-logarithmic phase was reached.

Hydrogen production experiments were conducted using a non-growing *Rhodospirillaceae* medium—modified minimal media containing (per liter): 0.6 g K_2_HPO_4_, 1.7 g KH_2_PO_4_, 0.02 g, MgSO_4_.7H_2_O, 0.005 g CaCl_2_.2H_2_O, 0.4 g NaCl, 0.3 g Na_2_S_2_O_3_, 0.0005 g ferric citrate, 0.0002 g para-aminobenzoic acid, and 1 mL of trace element solution containing: (per liter) 70 mg ZnCl_2_, 100 mg MnCl_2_.4H_2_O, 60 mg H_3_BO_3_, 200 mg CoCl_2_.6H_2_O, 20 mg CuCl_2_.2H_2_O, 20 mg NiCl_2_.6H_2_O, and 40 mg NaMoO_4_.2H_2_O [44]. The minimal media was autoclaved at 121 °C for 20 min, and the pH of the medium was measured at approximately 7.2 after autoclaving. To the medium was added 1 mL of a vitamin solution, consisting of (per liter): 1.2 g thiamine HCl and 0.01 g cyanocobalamin, filtered sterilized, as well as 10 mL of 5M sterile glycerol, to give a final glycerol concentration of 50 mM [44]. This medium, without any nitrogen sources, was formulated for the stationary phase (non-growing) production of hydrogen by *R. palustris cells*. Precultured cells were centrifuged in autoclaved 250 mL centrifuge bottles (Nalgene) at 3500× *g* for 15 min (HERMLE Labortechnik GmbH, Wehingen, Germany, Z366), after which the supernatant was removed aseptically inside a sterile laminar flow cabinet, and the cell pellets were washed and resuspended in sterile minimal media. This procedure was repeated three times to ensure that no Van Niels media, and therefore, also no nitrogen remained on the cell pellets—this was to ensure a constant biomass concentration throughout each experimental run, i.e., no growth of *R. palustris*. After washing, the cell pellets were resuspended in sterile non-growing minimal media. Chemically pure, biological grade glycerol (glycerol CP, 99% assay, Science World ©, Cape Town, South Africa) was used throughout in this study.

### 2.2. Photobioreactor Setup

The laboratory-scale prototype TPBR comprised a glass tubular loop (diameter of 24 mm), a cooling water jacket, and a GL45 polypropylene lid at the top of the reactor, which was modified by adding gastight stainless steel sampling ports. The riser section (length of 600 mm) was illuminated by a bank of four halogen floodlights (Eurolux ©, FS13, 150 W), while the rest of the reactor, including the downcomer section (length of 450 mm) was insulated and shaded from the light. Cooling water was pumped from a water chiller (model F25, Julabo GmbH, Seelbach, Germany), circulated through a cooling water jacket (volume of 442 mL) on the reactor and returned to the chiller at a fixed flow rate of 0.5 L min^−1^. It should be noted that the chiller was only used for the prototype TPBR—to reduce all costs associated with the TPBR, a cooling system operating without external energy inputs, e.g., cooling fins, will be employed in the future.

**Figure 1:**
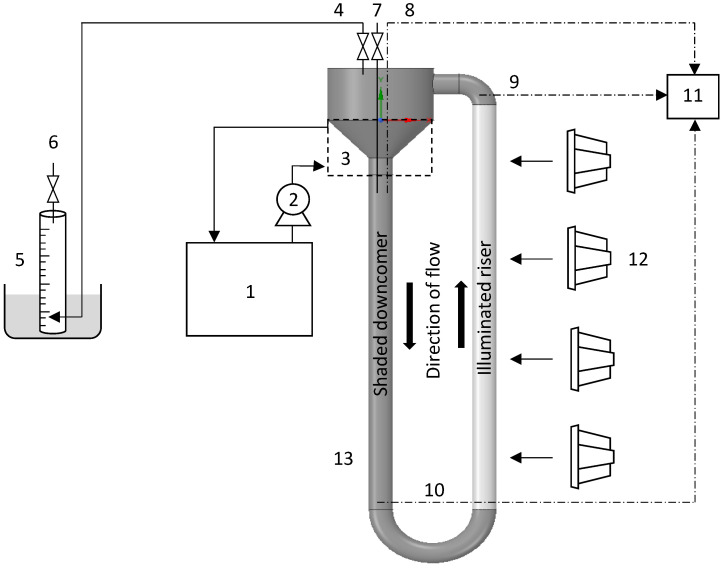
Schematic representation of the thermosiphon photobioreactor used for hydrogen production: (1) Water chiller; (2) Cooling water pump; (3) Cooling water jacket; (4) Gas collection port; (5) Inverted measuring cylinder submerged in water bath; (6) Gas sampling port; (7) Liquid sampling port; (8) Temperature probe 1; (9) Temperature probe 2; (10) Temperature probe 3; (11) Data logging unit; (12) Light source; (13) Photobioreactor.

Evolved gas was collected in an inverted measuring cylinder (1 L), immersed in a water bath. The cylinder was connected to the PBR through low hydrogen-permeability tubing (Tygon E-3603, Saint Gobain, Midrand, South Africa) which was fitted with a one-way valve to prevent reverse flow into the reactor. The volume of evolved gas was quantified via the water displacement method. For gas analysis, samples were taken from the gas sampling port situated at the top of the gas collecting chamber. Liquid samples (biomass and glycerol concentration) were taken aseptically from the liquid sampling port at the top of the reactor. The reactor temperature was monitored using three strategically positioned temperature sensors (3-wire PT100, 3 mm diameter, stainless steel sheath) connected to a data logging system. Figure 1 shows the experimental set-up.

### 2.3. Experimental Procedure

A specified concentration of *R. palustris* cells suspended in 1 L of modified minimal media was aseptically added to an autoclaved (121 °C, 20 min) TPBR. The reactor was sparged with filter sterilised (Midisart^®^ 2000 PTFE filter, 50 mm diameter, 0.2 µm pore size) argon gas (>99.9%) for 10 min, to ensure a dinitrogen-free atmosphere required for hydrogen production. The experimental run was initialized by switching on the halogen floodlights and cooling water to the reactor. Liquid samples were taken in time intervals of approximately 24 h, over a duration of 208 h. Similarly, the volume of evolved gas was also noted approximately every 24 h. All experimental runs were conducted in batch-mode, following the Box–Behnken experimental design with three center-point replications, allowing for the determination of statistical significance and standard deviation.

### 2.4. Analytical Methods

To determine the cell dry weight (CDW), a CDW versus optical density (OD) standard curve was developed. OD measurements were made using a UV/Vis-spectrophotometer (Model AE-S60-4U), and converted to CDW using the following correlations: CDW = 0.7126 × OD_660nm_ − 0.007 (Van Niels medium), *R*^2^ = 0.9981; CDW = 0.6391 × OD_660nm_ + 0.0619 (minimal medium), *R*^2^ = 0.9996. The concentration of glycerol in the samples was measured using high-performance liquid chromatography (Dionex UltiMate 3000 HPLC). Samples were passed through disposable syringe filters (FilterBio^®^ Nylon Syringe Filter, 13 mm diameter, 0.22 µm pore size) to remove all solid particles and to avoid blocking the HPLC column. Samples were then injected into the HPLC column (Bio-Rad Laboratories Ltd., Johannesburg, South Africa, HPX-87H column, 250 × 7.8 mm with guard cartridge) operating at a temperature of 65 °C, using an ERC Refracto Max520 RI detector. The mobile phase in the HPLC was a 0.005M H_2_SO_4_ solution at a flow rate of 0.6 mL min^−1^. Evolved gas samples were taken with a gastight gas sampling syringe, and analyzed using a gas chromatograph (Global Analyser Solutions Compact Gas GC). The GC was equipped with a thermal conductivity detector (110 °C), using packed columns (Rt-QBond, 3 m × 0.32 mm and Molsieve 5A 3 m × 0.533 mm). Argon was used as the carrier gas (45 kPa), using 50 µL injections at 60 °C, with a split of 5 mL min^−1^. The oven temperature was set to 65 °C, the filament temperature was at 210 °C, and a reference flow rate of 1 mL min^−1^ was used. Since *R. palustris* only produces H_2_ and CO_2_, other gases present in the gas samples were not taken into account, and the GC values were normalized for H_2_ and CO_2_. The PBRs were illuminated by halogen flood lights. The light intensity was measured using a handheld spectrometer (RGB Photonics, Qmini VIS-NIR) with an optical fiber probe.

### 2.5. Theory and Calculations

RSM is a useful tool for the investigation of the effect of specific independent factors on a response, as well as for the investigation of the interaction between certain independent factors [45,46]. For RSM and the fitting of second-order regression models, a Box–Behnken design of experiments with center-point replications is typically preferred above other designs [47]. This design effectively reduces the number of experiments while still providing sufficient data for evaluation of the complete system [47]. In this study, a Box–Behnken design with three factors and three center-point replications was implemented together with RSM. Using the MATLAB (R2021a) software package, quadratic polynomial regression models (Equation (1)) were developed to predict the response in the (i) rate of hydrogen production per reactor volume (mol H_2_ m^−3^_reactor_h^−1^); (ii) the specific rate of hydrogen production (mmol H_2_ g_CDW_^−1^h^−1^); (iii) the hydrogen yield (%); (iv) the substrate consumption (%); and (v) the biomass suspension (%).

(1) $$Y_{x}=\beta_{0}+\sum_{j}\beta_{j}x_{j}+\sum_{j}\beta_{jj}x_{j}^{2}+\sum_{i<j}\beta_{ij}x_{i}x_{j}$$

In Equation (1), *Y* denotes the response parameter, *β*_0_ is the offset term, *β_j_* and *β_jj_* the linear and quadratic coefficients, respectively, *β_ij_* the interaction coefficient, and *x* the independent input variables. Table 1 summarizes the independent input variables used in the experimental design.

The ranges chosen for the input values were based on previous work on the batch photofermentation of glycerol using *R. palustris* [6,12,48,49], and on preliminary work on a prototype TPBR [30,43], keeping in mind the physical constraints of both the bacterial species as well as the reactor geometry. The light intensity range was chosen based on the light intensity range in which *R. palustris* has been shown to grow and produce hydrogen. As mentioned, this range is approximately 70 to 600 W m^−2^ [6]; however, *R. palustris* has been shown to be more productive in the higher end of this range. Though it would have been interesting to see what the response surfaces would have looked like when extending the range to the lower end (closer to the minimum of 70 W m^−2^), such low light intensities would not have been of much interest in terms of hydrogen productivity by *R. palustris*, as photo-saturation has been shown to start at approximately 200 W m^−2^ [50]; therefore, the use of such low light intensities was decided against. The temperature range was chosen so as to achieve an operating temperature range inside the reactor that fell within the physiological limits of *R. palustris*. The cells produce hydrogen in the range of 30 to 42 °C [12], and they start to die when exposed to temperatures beyond 42 °C [51]. The biomass concentrations were chosen mainly based on light attenuation and hydrogen productivity. Concentrations lower than 0.4 kg m^−3^ would have been relatively low for sufficient hydrogen production in the reactor, while concentrations greater than 1.2 kg m^−3^ would have resulted in all the light being attenuated through the cross-section of the riser of the reactor. As a result, the rear-end of the riser section would have been in the dark, essentially being a dead zone with no productivity.

To test the statistical significance of the regression models, an analysis of variance (ANOVA) was conducted. For each regression model, the *R*^2^ and adjusted *R*^2^-values, together with the model’s *p*-statistic, are given. A model exhibiting *R*^2^-values greater than 0.95 shows a good fit to the experimental data, while a *p*-value of less than 0.05 suggests a statistically significant correlation between the model/independent variable and the response [47]. Regression models were reduced, based on the statistical significance criterion where terms in the models having *p*-values greater than 0.05 were eliminated to produce models consisting only of statistically significant terms.

The rate of hydrogen production was assessed, both in terms of the reactor working volume (*V*) (Equation (2)), and the biomass concentration (*m*) (Equation (3)), using the total amount of hydrogen, in moles (Δ*n*), produced over the course of the experimental run, together with the final time of 208 h (*t_final_*).

(2) $${\mathrm{Rate}\;\mathrm{of}\;H}_{2}\;\mathrm{production}\;\left[\;{\mathrm{mol}\;m}^{-3}h^{-1}\right]=\frac{\Delta n_{H_{2}\;\mathrm{measured}}}{Vt_{\mathrm{final}}}$$

(3) $${\mathrm{Rate}\;\mathrm{of}\;H}_{2}\;\mathrm{production}\;\left[{\mathrm{mmol}\;g}_{\mathrm{CDW}\;}^{-1}h^{-1}\right]=\frac{\Delta n_{H_{2}\;\mathrm{measured}}}{mt_{\mathrm{final}}}$$

Hydrogen yield (Equation (4)) was determined as the molar ratio of hydrogen produced to glycerol, consumed as a percentage of the theoretical maximum, per the stoichiometric conversion of glycerol to hydrogen: C_3_H_8_O_3_ + 3H_2_O → 3CO_2_ + 7H_2_. The molar volume of hydrogen (at NTP) was determined using the composition of hydrogen in the evolved gas. The hydrogen content in the evolved gas varied between 88% and 94% (±1%), with the balance being carbon dioxide.

(4) $$H_{2}\;\mathrm{yield}\;\left[\%\right]=\frac{\Delta n_{H_{2}\;\mathrm{measured}}}{7\Delta n_{\mathrm{glycerol}\;\mathrm{consumed}}}\times 100$$

Glycerol consumption was evaluated as the molar ratio of glycerol consumed at time *t*, to the glycerol initially in the system (Equation (5)).

(5) $$\mathrm{Glycerol}\;\mathrm{consumed}\;\left[\%\right]=\frac{n_{o,\mathrm{glycerol}}-n_{t,\mathrm{glycerol}}}{n_{o,\mathrm{glycerol}}}\times 100$$

Biomass suspension was assessed as the ratio of the concentration of bacterial cells in free suspension at time *t* (*c_t_*), to the initial biomass concentration (*c_o_*) measured before each experimental run (Equation (6)). A liquid sample was taken at the top of the reactor to determine the biomass concentration in free suspension at any time, while the initial biomass concentration remained constant throughout each experimental run, due to the use of non-growing culture medium—this was verified by also measuring the biomass concentration at the end of each run.

(6) $$\mathrm{Biomass}\;\mathrm{in}\;\mathrm{suspension}\;\left[\%\right]=\frac{c_{t}}{c_{o}}\times 100$$

Table 2 summarizes the experimental design, together with corresponding results for the coded input variables. The coded variable −1 refers to the smallest value, 0 the midpoint value, and 1 the largest value for the independent input variables described above. Since the measured biomass concentrations slightly deviated from the three specified levels, the input values for this predictor variable were recoded according to Equation (7), using the experimentally measured values.

(7) $$x_{3,\mathrm{recoded}}=\frac{\left(x_{3,\mathrm{measured}}-\left(\frac{x_{3,\max}-x_{3,\min}}{2}+x_{3,\min}\right)\right)}{\frac{\left(x_{3,\max}-x_{3,\min}\right)}{2}}$$

The standard deviations of the time profiles referred to in Section 3.1 and Section 3.3 were determined from the center-point replication runs and extended over the entire data set at all conditions. As mentioned above, the initial biomass concentrations at the center-points from which standard deviation was calculated were not exactly equal, with small differences between the three values (<0.043 g L^−1^)—this is expected to have had a slight effect on the calculated standard deviations; however, the standard deviation was still reported to give a good guideline of the variance in the data.

## 3. Results & Discussion

### 3.1. Rate of Hydrogen Production

To determine the effects of the mentioned operating parameters, predictive models were developed for response parameters, such as rate of hydrogen production (Equations (8) and (9)). The model generated for the rate of hydrogen production per reactor volume fitted the experimental data well, with *R*^2^- and adjusted *R*^2^-values of 0.969 and 0.957, respectively, and an overall *p*-value of 1.63 × 10^−7^. The prediction model for specific hydrogen production per biomass concentration had *R*^2^- and adjusted *R*^2^-values that were slightly lower −0.893 and 0.850, and a *p*-value of 0.0000767.

**Figure 2:**
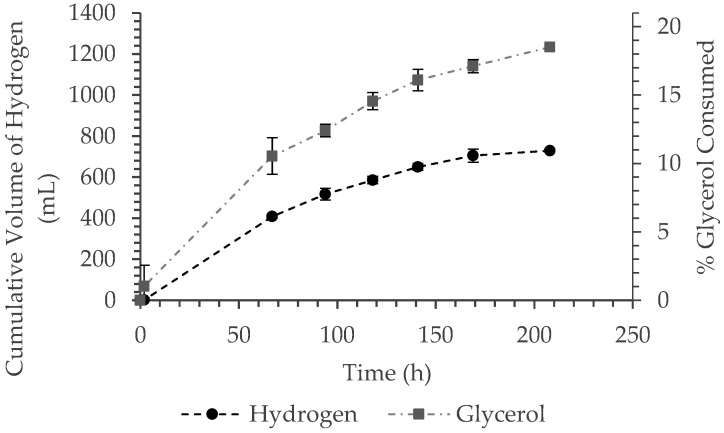
Time profiles of cumulative hydrogen production and glycerol consumption (cooling water temperature of 21 °C, light intensity of 500 W m^−2^, and biomass concentration of 1.22 g L^−1^).

The rate of hydrogen production was affected both by biomass concentration as well as cooling water inlet temperature and the interaction between biomass concentration and light intensity. The actual operating temperature inside the TPBR, defined as the average of the three temperatures measured in the reactor (described in Section 2), was in the range of 31 to 44 °C, depending on the operating conditions, but no trend was seen with regards to combinations of operating conditions and the operating temperature in the reactor. As mentioned, du Toit has shown *R. palustris* to produce hydrogen in the temperature range of 30 to 42 °C [12]. According to the prediction models, a maximum production rate per volume of 0.16 mol m^−3^h^−1^ can be achieved at a biomass concentration of 1.16 g L^−1^. This was expected, as more bacterial cells were present in the reactor to produce hydrogen, while the reactor working volume remained constant. An experimentally measured time-profile of the cumulative hydrogen production at similar conditions to that predicted with the response model (Equation (8)) is provided in Figure 2. Here, the mathematically predicted rate of hydrogen production compares well with the experimentally measured value of 0.156 mol m^−3^h^−1^ at similar conditions.

Conversely, the regression model predicted a maximum production rate per biomass concentration of 0.21 mmol g_CDW_^−1^h^−1^ at a light intensity of 600 W m^−2^ and biomass concentration of 0.40 g L^−1^. At this biomass concentration, the model predicted a maximum rate of hydrogen production, which also compares well with the experimentally measured value of 0.185 mmol g^−1^h^−1^.

Rate_coded_ (mol m^−3^h^−1^) = 0.134 + 0.0358*x*_3_ + 0.0108*x*_2_^2^ − 0.0287*x*_3_^2^ − 0.00873*x*_1*x*3_ (8)

Rate_coded_ (mmol g_CDW_^−1^h^−1^) = 0.160 − 0.0355*x*_3_ + 0.0179*x*_2_^2^ − 0.0166*x*_3_^2^
*−* 0.0132*x*_1_*x*_3_ (9)

**Figure 3:**
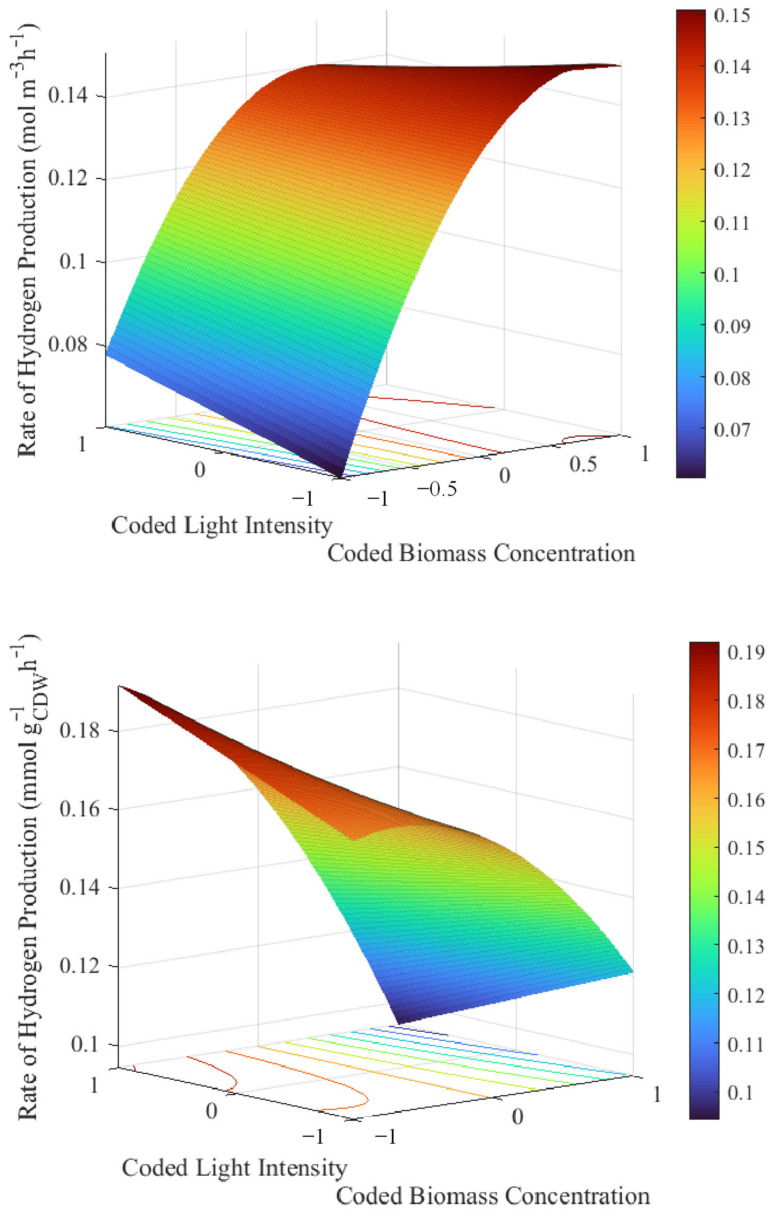
Response surface plots for the rate of hydrogen production per (**top**) reactor working volume; and (**bottom**) initial biomass concentration (models were plotted at the midpoint value of the third predictor variable not displayed on the graphs, i.e., at a cooling water inlet temperature of 19 °C).

The hydrogen production rate per biomass concentration was highest at the highest light intensity, and the lowest biomass concentration in the ranges evaluated (Figure 3). This light intensity of 600 W m^−2^ compares well with the average maximum natural solar light intensity of approximately 550 W m^−2^, as measured in Stellenbosch, South Africa, over a 7 day period in the month of May [6]. This suggests that the TPBR should be able to achieve similar hydrogen production rates under outdoor conditions; however, slightly lower production rates are expected early in the morning during periods of lower light intensities.

This finding also coincides with a study by du Toit on the heat acclimation of *R. palustris* cells [12]. According to the Beer–Lambert law of light attenuation, the light intensity would be attenuated by approximately 86% and 99% for *R. palustris* concentrations of 0.40 g L^−1^ and 1.25 g L^−1^, respectively, in the TPBR riser section with a cross-sectional diameter of 24 mm. From a visual observation of the rising velocity of the bacterial cells, it was noted that the riser section of the TPBR presented with slightly stratified flow patterns, which meant that the rear-end of the riser section had upward velocities that were substantially lower than the illuminated front-end of the riser section. As a result, the bacterial cells at the rear-end of the riser section spent long periods of time under conditions of little to no light at a higher biomass concentration of 1.25 g L^−1^—an issue that could be circumvented by introducing axial mixing structures into the riser section or a strategy for enhanced light distribution. A specific rate of hydrogen production being higher at low biomass concentrations and high light intensities was therefore expected, as this combination of conditions would result in diminished light attenuation in the reactor. Consequently, more bacterial cells would be exposed to higher light intensities, resulting in the optimal ATP regeneration necessary for the production of hydrogen [12]. 

The hydrogen production rates achieved in the TPBR were comparably lower than the rates achieved with *R. palustris* in more conventional PBRs with external mixing/circulation (Table 3). Due to the passive circulation nature of the TPBR, this is expected, as some bacterial cells, specifically the larger immotile mother cells, will settle out over time, while most cells are expected to remain in suspension in bioreactors with constant external mixing. Additionally, only half of the TPBR is illuminated, essentially halving the illuminated working volume, and therefore, also the concomitant hydrogen produced in such a reactor, as compared to a PBR that is always fully illuminated. Nonetheless, by eliminating pumping/mixing, the operating cost of the TPBR would also be lower than for externally mixed PBRs.

Due to light intensity and the cooling water temperature having little to no effect on the efficiency of the proposed system, the number of operating conditions can be reduced for the optimization of the system, simplifying the process. Furthermore, because the ultimate aim of the proposed photobioreactor is to operate with little to no external energy inputs, the light intensity and cooling water temperature having little effect on the productivity of the system could be seen as being advantageous when aiming to reduce energy inputs and operate under outdoor conditions.

### 3.2. Glycerol Consumption

From experimental measurements, between 8 and 19% (±0.48%) of the initial glycerol in the system (50 ± 4.3 mM) was consumed by the *R. palustris* cells. The regression model for glycerol consumption had *R*^2^- and adjusted *R*^2^-values of 0.769 and 0.706, respectively, which was quite low (Equation (10)). The model had an overall *p*-value of 0.0008, suggesting a statistically significant prediction model.

% Glycerol consumed_coded_ = 13.114 + 3.393*x*_3_ + 3.096*x*_2_^2^ − 2.251*x*_3_^2^ (10)

In the range of 400 to 600 W m^−2^ (±20 W m^−2^), the light intensity had no statistically significant effect on glycerol consumption. This was also the case for the cooling water inlet temperature, while the initial biomass concentration had the most significant effect. The optimal biomass concentration for glycerol consumption by *R. palustris* was approximately 1.15 g L^−1^, predicting a glycerol consumption of 17.5% of the initial concentration in the system.

### 3.3. Hydrogen Yield

Of the 8 to 19% of glycerol that was consumed during each experimental run, approximately 45 to 77% (±3.8%) of that glycerol was converted to hydrogen gas, depending on the conditions—this was slightly lower than the literature-reported crude glycerol conversion efficiency of 90% [7]. The regression model constructed for hydrogen yield (Equation (11)) did not fit the experimental data well, with an *R*^2^-value of 0.441 and an even lower adjusted *R*^2^-value of 0.348. The reduced prediction model produced a *p*-value of 0.030.

%H_2_ Yield_coded_ = 59.854 + 5.763*x*_2_ − 7.831*x*_3_^2^ (11)

In the ranges investigated, the experimentally determined hydrogen yield was relatively low [8,15] prompting further investigation into the time profiles of hydrogen production and glycerol consumption (Figure 2). From the time profiles of hydrogen production, it can be seen that the cumulative hydrogen production slowly started to plateau after approximately 144 h, while this was not the case for the glycerol consumption. The continued consumption of glycerol after hydrogen production started to decrease, suggests that the glycerol was being directed elsewhere. HPLC analysis was conducted to test for the production of waste by-products. Waste by-products, more specifically acetic acid, butyric acid, ethanol, and butanol, are produced through the incomplete oxidation of glycerol (Equations (12)–(15)), and typically occurs during dark fermentation [49]; however, none of these compounds were found to be present in the system.

C_3_H_8_O_3_ + H_2_O → CH_3_OOH + CO_2_ + 3H_2_ (12)

2C_3_H_8_O_3_ → C_4_H_8_O_2_ + 2CO_2_ + 4H_2_ (13)

2C_3_H_8_O_3_ → C_4_H_10_O + 2CO_2_ + H_2_O + 2H_2_ (14)

C_3_H_8_O_3_ → C_2_H_6_O + CO_2_ + H_2_ (15)

**Figure 4:**
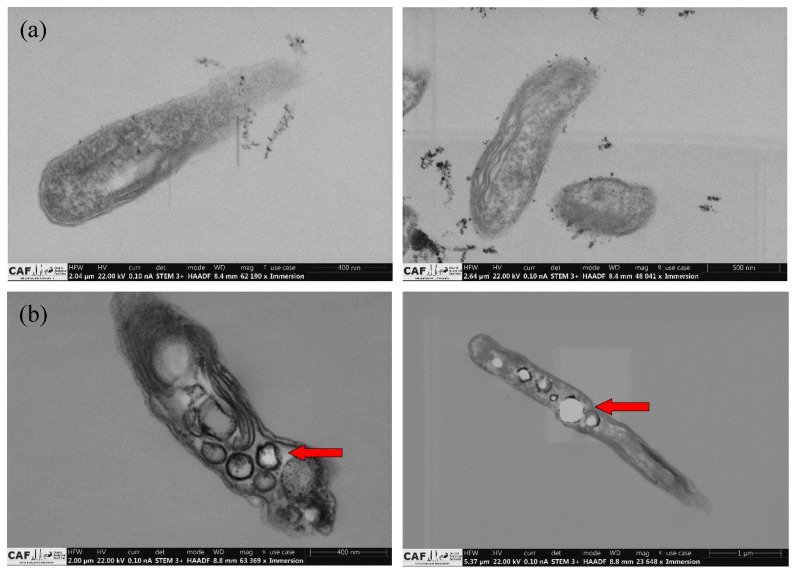
Scanning transmission electron microscopy images for *R. palustris* cells (**a**) before and (**b**) after experimental runs (PHB granules indicated by red arrows)—images taken by the Central Analytical Facilities (CAF) of Stellenbosch University.

It has also been shown that *R. palustris* tends to generate internal storage products, specifically poly-hydroxybutyrate (PHB), glycogen, and trehalose [54,55] when subjected to suboptimal conditions such as nutrient starvation [56,57]. Since the bacterial cells were starved of nitrogen for the experiments in this study, scanning transmission electron microscopy images (Figure 4) were taken to determine whether glycerol had been partially directed towards PHB production. The white circles (PHB granules) present in the bacterial cells after the experimental runs, in contrast to the absence of white circles in the bacterial cells before the experimental runs, strongly suggests the presence of internal PHB granules stored as reserves for the survival of the cells. This would then explain the low glycerol to hydrogen conversion and what the remainder of the utilized glycerol had been used for.

### 3.4. Biomass Suspension

The percentage of bacterial cells in suspension decreased substantially over the duration of the experimental runs, with the final measured suspension values (at 208 h) ranging from approximately 42 to 75% (±2.9%), depending on the conditions. From visual observation, the cells seemed to settle on all horizontal/inclined areas, presumably due to insufficient fluid velocity. The predictive model for biomass suspension is given by Equation (16). The model produced an *R*^2^-value of 0.843, an adjusted *R*^2^-value of 0.800, and a *p*-value of 0.0000998, indicating a statistically significant regression model.

% Suspension_coded_ = 51.653 − 10.077*x*_3_ − 6.798*x*_2_^2^ + 10.606*x*_3_^2^ (16)

**Figure 5:**
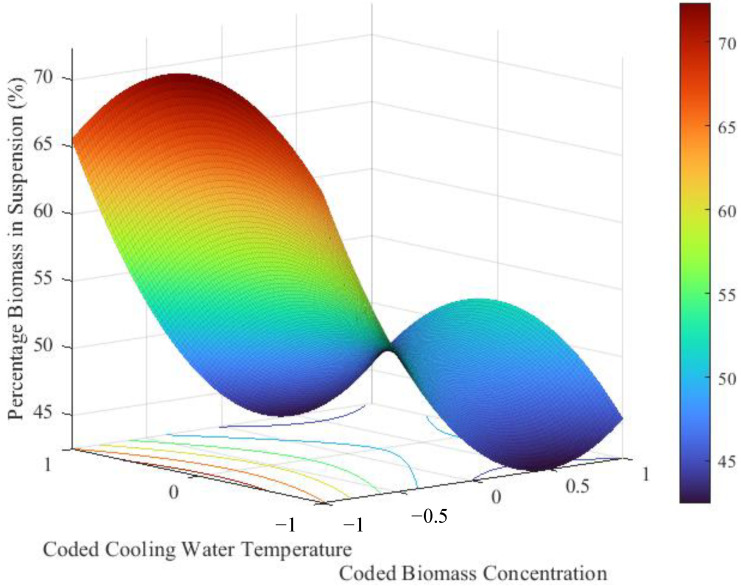
Response surface plot for the percentage biomass in suspension (model plotted at the midpoint value of the third predictor variable not displayed on the graph, i.e., at a light intensity of 500 W m^−2^).

As for glycerol consumption and hydrogen yield, the light intensity did not have a statistically significant effect on the suspension of biomass in the reactor, while biomass concentration had the most pronounced effect. According to the prediction model, as well as the response surface plot provided in Figure 5, the maximum percentage of biomass in suspension will be achieved at a biomass concentration of 0.40 g L^−1^, the lowest concentration in the range investigated, maintaining approximately 72.3% of the biomass in suspension over a period of 208 h.

A biomass circulation being better at lower biomass concentrations was to be expected; however, the measured suspension values were still relatively low. This can be attributed to the upward circulation velocity in the reactor being slower than the terminal settling velocities of the bacterial cells, and/or clumps of cells forming. It is also inferred that the cells in suspension had mostly been motile daughter cells, rather than the larger mother cells. The daughter cells are smaller than the mother cells [58], meaning that they have slower terminal settling velocities and would require a slower upward circulation velocity to maintain them in suspension. Daughter cells also have flagella, which make them motile, while the larger mother cells are not motile [59]. As a result, the larger mother cells tend to settle out faster than the motile daughter cells. Furthermore, in the event of the *R. palustris* cells being under sub-optimal conditions—e.g., nutrient starvation, mutual shading, excessive light intensity, inefficient mixing, and/or operating temperatures outside the physiological limits of the cells—the cells tend to lump together, forming small clumps with higher terminal settling velocities than for single bacterial cells [60]. This is a phenomenon that has been observed visually, both in the currently proposed TPBR as well as in previous preliminary work using Shott bottles under controlled conditions. To maintain the bacterial cells in suspension, the operating conditions and the geometry of the TPBR should therefore be adjusted in order to increase the circulation velocity.

## 4. Conclusions

In this work, hydrogen production experiments were conducted at various independent operating conditions to evaluate the use of a prototype TPBR for photofermentative hydrogen production by *R. palustris*. The effects of light intensity, inlet cooling water temperature, and biomass concentration were evaluated with RSM. The predictive regression models generated were used to investigate the effect of the abovementioned operating conditions on hydrogen production, as well as biomass circulation in the proposed TPBR.

Biomass concentration was found to have had the most significant effect on the rate of hydrogen production and glycerol consumption, as well as biomass suspension. The effect of light intensity was expected to be more pronounced; however, it was only significant for the rate of hydrogen production when interacting with biomass concentration. Further investigations into light intensity and light distribution in the TPBR would be beneficial to the understanding of this system. The inlet cooling water temperature had little effect on the evaluated responses.

In the ranges investigated, the proposed TPBR generated satisfactory fluid flow and was able to maintain up to 77% of biomass in suspension. The TPBR itself performed best when containing lower concentrations of *R. palustris* cells, i.e., approximately 0.4 g L^−1^; however, in terms of maximum hydrogen production and carbon substrate consumption, the overall system performed better at higher biomass concentrations of approximately 1.2 g L^−1^. The system was able to utilize approximately 8 to 19% of the carbon substrate present. Under the conditions investigated, *R. palustris* converted approximately 45 to 77% of the glycerol to hydrogen gas, while redirecting a portion of the consumed glycerol to the production of PHB. Though not the focus of the present study, PHB has gained increasing attention in the field of bioplastics, and it can therefore also add value to this proposed system, allowing for further investigation. For bioremediation and hydrogen production, it is recommended that the TPBR be operated at higher light intensities and biomass concentrations, provided that these conditions are within the physiological limits of *R. palustris*. Collectively, the overall research hypothesis of the study was verified—*R. palustris* cells were demonstrated to be able to produce hydrogen in the proposed TPBR, while, with a few alterations, the TPBR has also been proven to be a suitable prospect for the application of photofermentative hydrogen production. Though the hydrogen productivity of the system were slightly lower than that achieved by conventional photobioreactors, the proposed photobioreactor still merits consideration as an alternative photobioreactor for sustainable biohydrogen production—it is currently the only photobioreactor with the prospect of operating without any external energy inputs, which could, in the future, balance out its lower efficiency of hydrogen productivity.

## Figures and Tables

**Table 1:** Symbols and intervals used in response surface methodology.

Independent Variable	Symbol	Intervals		
		**−1**	**0**	**1**
Light Intensity (W m^−2^)	*x*_1_	400	500	600
Cooling Water Inlet Temperature (°C)	*x*_2_	17	19	21
Biomass Concentration (g L^−1^)	*x*_3_	0.40	0.82	1.25

**Table 2:** Box–Behnken experimental design with three independent variables.

Run	Coded Values			Experimental Results				
	*x*_1_	*x*_2_	*x*_3_	Rate of H_2_ Production (mol m^−3^h^−1^)	Rate of H_2_ Production (mmol g_CDW_^−1^h^−1^)	% H_2_ Yield	% Glycerol Consumed	% Biomass in Suspension
1	−1	0	−0.927	0.063	0.147	48.7	9.45	74.36
2	1	0	−0.984	0.076	0.185	57.6	8.48	73.72
3	0	0	−0.189	0.128	0.171	60.7	12.8	53.33
4	−1	−1	−0.259	0.123	0.178	48.1	14.4	42.49
5	1	−1	−0.184	0.145	0.194	45.2	18.8	48.08
6	0	−1	−1.00	0.081	0.201	50.5	9.83	58.14
7	1	0	1.00	0.134	0.107	53.4	13.7	47.80
8	0	0	0.009	0.128	0.154	58.2	12.1	47.64
9	−1	0	0.960	0.149	0.121	60.5	12.9	56.46
10	−1	1	−0.075	0.132	0.166	64.3	12.2	51.80
11	1	1	−0.085	0.142	0.180	77.1	17.3	49.46
12	0	−1	0.968	0.148	0.119	48.9	18.5	45.96
13	0	0	−0.111	0.140	0.179	65.7	13.0	50.95
14	0	1	0.921	0.156	0.128	45.9	18.5	43.46
15	0	1	−0.979	0.088	0.215	53.3	9.09	67.37

**Table 3:** Comparison of hydrogen production rates of various *R. palustris* strains in other photobioreactors.

Reactor Type	Strain	H_2_ Production Rate (mol m^−3^h^−1^)	Reference
Biofilm PBR	*R. palustris* CQK01	1.74	[8]
Biofilm PBR	*R. palustris* CQK01	1.75	[52]
Optical fibre PBR	*R. palustris* WP 3-5	1.96	[15]
Glass bottle PBR	*R. palustris* DSM 127	1.23	[53]
Tubular PBR	*R. palustris* 420 L	1.20	[16]
Glass bottle PBR	*R. palustris* GCA009	0.72	[12]
Glass bottle PBR	*R. palustris* ATH 2.1.37	0.98	[12]
Thermosiphon PBR	*R. palustris* NMIB1774	0.16	Present study

## Data Availability

Not applicable.
